# Efficacy of Mating Disruption Treatments Against Spongy Moth (*Lymantria dispar dispar*) Applied Using Unmanned Aerial Vehicles

**DOI:** 10.3390/insects17060650

**Published:** 2026-06-20

**Authors:** Ksenia S. Onufrieva, Andrea D. Hickman, Tom W. Coleman

**Affiliations:** 1 Department of Entomology, Virginia Tech, Blacksburg, VA 24061, USA; anhickma@vt.edu; 2 USDA Foreign Agricultural Service, Global Programs, Asheville, NC 28801, USA; tom.coleman@usda.gov

**Keywords:** spongy moth, *Lymantria dispar*, mating disruption, SPLAT, UAV, drone

## Abstract

Unmanned aerial vehicles (UAVs) are increasingly used in precision pest management, yet their performance in operational forest settings remains underexplored. We evaluated the efficacy of SPLAT^®^ SM-O mating disruptant applied using a UAV at a dosage of 14.8 for control of the spongy moth, *Lymantria dispar dispar* L. (Lepidoptera: Erebidae). One treatment plot received 11.4 g AI/ha because of a calibration deviation during application. Both treatments reduced trap catches by >90% for 10 weeks following the application, meeting the efficacy requirement set by the USDA’s National Slow the Spread (STS) Program. One year after the application, trap catches continued to be reduced by 28% and 67% in plots treated with 14.8 and 11.4 g AI/ha, respectively. These levels of trap catch reduction in the year of treatment and one year after the treatment application are comparable to those reported following fixed-wing aerial treatments. These results indicate that UAV-applied SPLAT^®^ SM-O meets STS requirements for operational use and is suitable for integration into the program for treating small or isolated blocks. These findings also have broader implications for the use of unmanned aerial vehicles to deploy SPLAT^®^ formulations in forest pest management programs.

## Simple Summary

The spongy moth is a destructive forest pest that is managed in the United States through the Slow the Spread (STS) Program. One of the program’s primary management tools is mating disruption, which uses synthetic pheromones to prevent male moths from locating females and reproducing. Traditionally, these treatments are applied using fixed-wing aircraft. However, conventional aircraft may be difficult or uneconomical to use in small, isolated, or fragmented treatment areas. In this study, we evaluated the effectiveness of applying a pheromone-based mating disruption treatment using unmanned aerial vehicles (UAVs, or drones). Treatment performance was assessed using standard STS monitoring methods and compared with established program criteria. UAV-applied treatments provided effective suppression of male moth captures during the year of application and showed persistence into the following year, with results comparable to those reported for conventional aerial applications. These findings demonstrate that UAVs can successfully deliver operational mating disruption treatments without compromising efficacy. UAV-based applications may provide managers with a flexible tool for treating areas that are difficult to access using conventional aircraft and could help expand the use of pheromone-based pest management in forest ecosystems.

## 1. Introduction

Unmanned aerial vehicles (UAVs) are increasingly being used in pest management, but their performance under operational conditions, particularly in forested systems, remains underexplored. Combining a UAV with a precise application payload is expected to provide a safer, more economical method of aerial treatment application with minimized off-target movement of the sprayed agent [1]. Piloted aircraft are limited by high operational complexity and cost [2], are unsuitable for small and/or fragmented areas, and are shown to fail in small treatment blocks due to inadequate pheromone coverage [3]. As a result, pest management programs are frequently unable to treat all areas in need of protection, while private growers and homeowners may resort to conventional insecticides that are potentially harmful to non-target organisms. Integrating UAV technology into pest management programs offers a promising solution by enabling more targeted applications and expanding treatment access to previously inaccessible areas. However, without clear guidance and field-based validation, the potential of UAVs may remain unrealized. As stated in the USDA’s National Roadmap for Integrated Pest Management [4], effective pest control requires the use of “available technology to prevent unacceptable levels of pest damage by the most economical means, while posing the least possible risk to people, property, resources, and the environment.” UAV applications can help achieve that goal only if their use is systematically tested, refined, and integrated into operational programs.

Many countries outside the United States have long used UAVs in agricultural applications; for example, Japan relies on UAVs for approximately 90% of all aerial applications for field crops [5]. In the U.S., recent field trials have begun to build a foundation for broader adoption by demonstrating that UAV-based pesticide applications can achieve efficacy comparable to that of conventional aerial applications [6] and ground-based sprayers [7]. In cotton production, Cavalaris et al. [8] found that UAV applications of defoliants were more efficient than both ground and aerially applied systems, highlighting the potential for UAVs to improve operational flexibility and reduce overall chemical use. Trials in cranberry systems have demonstrated that UAV-applied pheromones can significantly reduce trap catches and the mating success of two Lepidopteran pests [9]. Collectively, these studies support the growing role of UAVs as effective delivery platforms for pest management in agriculture, particularly in systems characterized by uniform crop structure and relatively simple canopy architecture.

Recent syntheses of UAV spray technology highlight that, despite substantial advances, key technical and ecological uncertainties remain unresolved. Zhang et al. [10] identify persistent challenges related to spray drift, canopy penetration, and spatial heterogeneity of deposition arising from complex interactions among droplet size, rotor downwash, canopy structure, and environmental conditions. Importantly, most existing UAV studies emphasize spray deposition metrics rather than biological efficacy or treatment persistence, particularly under operational field conditions. These limitations are expected to be amplified in forested environments with complex vertical structures and heterogeneous canopies.

Empirical evaluations of UAV applications remain limited in forested environments. Richardson [11] concluded that most UAV spray research has been conducted in agricultural systems and that forest canopies present fundamentally different challenges related to interception, deposition consistency, and operational feasibility. As a result, UAVs are unlikely to replace helicopters or fixed-wing aircraft for large-area broadcast treatments in forestry but may offer substantial advantages for precision applications in small, operationally constrained, or otherwise inaccessible treatment blocks. Supporting this perspective, Krasylenko et al. [12] demonstrated that small UAVs equipped with specialized manipulators can safely operate within complex forest canopies to perform highly localized inspection, sampling, and targeted spraying, highlighting the feasibility of precision UAV operations in structurally heterogeneous forest environments.

The present study addresses this gap by evaluating the efficacy and persistence of UAV-applied SPLAT^®^ SM-O (formerly SPLAT^®^ GM-Organic; ISCA, Riverside, CA, USA) for mating disruption of the spongy moth, *Lymantria dispar dispar* L. (Lepidoptera: Erebidae). The spongy moth is one of the most destructive invasive insects in North American forests. It is a federally regulated pest and managed through three strategies: suppression, eradication, and the National Slow the Spread (STS) Program [13]. STS is an area-wide integrated pest management program implemented at the leading edge of the expanding population front with the goal to reduce the rate of spongy moth spread across the United States. In the STS Program, mating disruption is the primary method of spongy moth control, and since 2017, SPLAT^®^ SM-O has been used exclusively on more than 100,000 ha annually, with an average treatment block size of 2029 ± 113 ha [14].

Additionally, given that SPLAT^®^ is a biologically inert matrix used in a range of semiochemical-based formulations designed for controlled release of an active ingredient [15,16,17,18,19,20], the results of this study may provide insights for other SPLAT-based management programs. Finally, while UAVs are often presented as a technological breakthrough in precision agriculture and pest management, some authors have cautioned against uncritical adoption of such narratives driven by media hype and inflated expectations [21]. Field-based trials—including this one—are therefore essential to evaluate the actual effectiveness of UAV-based treatments under operational field conditions, which can represent fluctuations in topography, isolated forest stands, limited sight lines, and varying forest canopy heights. Furthermore, the STS Program requires new methods for applying mating disruption treatments and the ability to treat small (~200 ha or smaller), isolated infestations.

## 2. Materials and Methods

### 2.1. Pheromone Formulation

**Figure 1:**
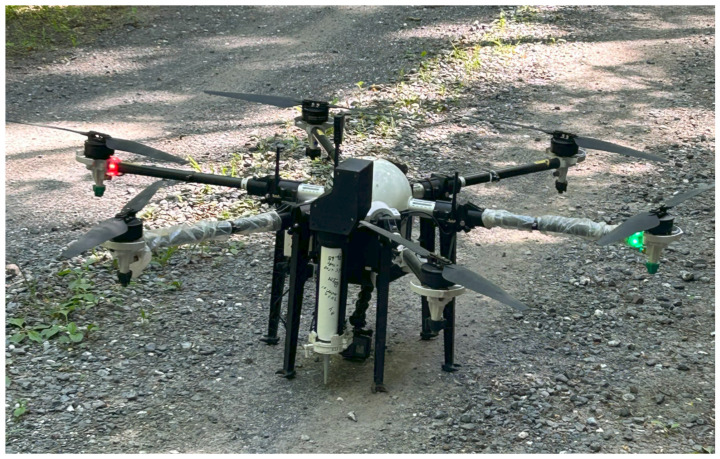
Unmanned aerial vehicle [agricultural drone sprayer M6E-X (Beijing TT Aviation Technology Ltd.)] equipped with Aksata (formerly Agridrones) Ointment Application System (white vertical tube) to apply SPLAT^®^ SM-O.

SPLAT^®^ SM-O, one of the formulations within the Specialized Pheromone and Lure Application Technology (SPLAT^®^) platform developed by ISCA Technologies (Riverside, CA, USA), was used as the mating disruption formulation in this study. SPLAT^®^ formulations are flowable, non-Newtonian, shear-thinning, thixotropic materials designed for the controlled release of semiochemicals in pest management [22,23]. Under mechanical stress (e.g., pumping or agitation), their viscosity decreases, allowing reliable delivery through mechanical application systems, while rapidly regaining structure once stress is removed. These properties enable SPLAT^®^ formulations to be applied using pressurized delivery systems while allowing deposited droplets to retain their shape and adhere to treated surfaces following application. After deployment, SPLAT^®^ formulations become rainfast within approximately three hours and can provide sustained release of incorporated semiochemicals for periods ranging from several weeks to several months, depending on formulation and environmental conditions. In this study, SPLAT^®^ SM-O, a formulation containing (+)-disparlure as the active ingredient at 13% (*w*/*w*), was applied using an unmanned aerial vehicle (UAV)-based delivery system (Figure 1).

### 2.2. Efficacy of the Mating Disruption Treatment in the Year of Application

**Figure 2:**
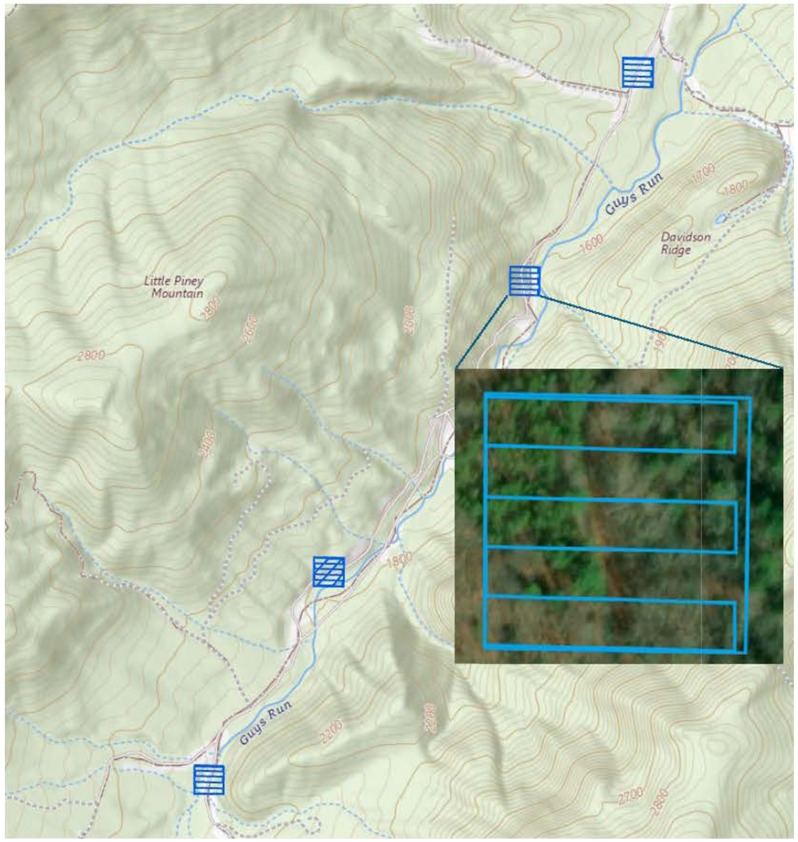
Experimental plots treated using the agricultural drone sprayer M6E-X (Beijing TT Aviation Technology Ltd.) equipped with Aksata Ointment Application System to apply SPLAT^®^ SM-O. Flightlines are shown in blue on the main map and in the inset.

Eight experimental plots, each 100 m × 100 m, were established in Goshen Wildlife Management Area, VA, within forest stands dominated primarily by oak (*Quercus* spp.) that had experienced minimal human disturbance or active forest management for over five years. Plots were established in either a valley bottom or along hillslope edges; however, all plots contained local elevational variation and were not arranged in a formal blocked design based on topography (Figure 2). Treatment and control plots were established within suitable forest stands that met operational and logistical requirements, with plots separated by at least 700 m to prevent interference among treatments [24].

Four plots were left untreated and used as a control, and four plots were treated on 26 May 2023 with SPLAT^®^ SM-O using an agricultural drone sprayer M6E-X (Beijing TT Aviation Technology Ltd., Beijing, China) equipped with an Aksata (formerly Agridrones, Kfar Saba, Israel) Ointment Application System (OAS) precise applicator payload (Figure 1). Three plots were treated with an overall dosage of 14.8 g/ha (6 g AI/acre), which is an operational dosage in the STS Program. For each plot, calibration was conducted immediately prior to treatment using standardized procedures for the UAV delivery system, including verification of formulation flow rate, flight speed, and programmed application volume. Each cartridge was weighed before and after application to determine the amount of product applied per plot. Based on cartridge weights measured before and after application, treated plots received 113.8, 110.3, 115.62, and 88.35 g of SPLAT^®^ SM-O, corresponding to application rates of approximately 14.8, 14.3, 15.0, and 11.4 g AI/ha, respectively.

One plot was treated with an overall dosage of 11.4 g AI/ha (4.6 g AI/acre) due to a calibration error during application. This plot was treated as an operational deviation and was retained in the study as a distinct application-rate case; it was analyzed separately and not pooled with plots treated at the target rate.

The drone flew at a speed of 10 m/s, 1.2 m (4 feet) above the canopy, rather than at a fixed altitude above ground level, treating plots at an average rate of 36 s per 0.4 ha (one acre). Although absolute canopy height varied among plots, this relative flight height ensured consistent clearance and delivery conditions across all treated areas.

**Figure 3:**
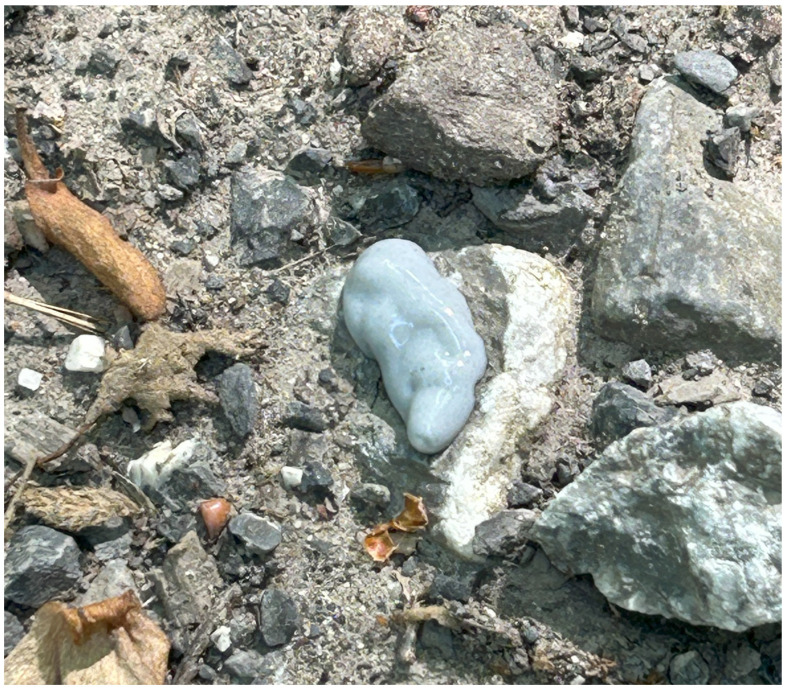
Large, cohesive masses of SPLAT^®^ SM-O deposited by UAV on target surfaces.

SPLAT^®^ SM-O was dispensed from a 250 g capacity cartridge compatible with a manual caulking gun. The cartridge was mounted within the Ointment Application System (OAS), a mechanical delivery system designed to dispense viscous formulations as discrete, non-atomized deposits rather than as a spray. During operation, mechanical pressure applied to the cartridge reduces formulation viscosity, enabling controlled extrusion through the nozzle, while the formulation rapidly regains structure after deposition. This delivery approach allows consistent placement of localized SPLAT^®^ deposits that adhere to treated surfaces and remain spatially confined following application. The product was extruded in a continuous line by steady pressure from the tube, with 15 m spacing between adjacent lines (Figure 2). During application, the material intermittently broke into large, cohesive masses that were deposited on target surfaces (Figure 3).

**Figure 4:**
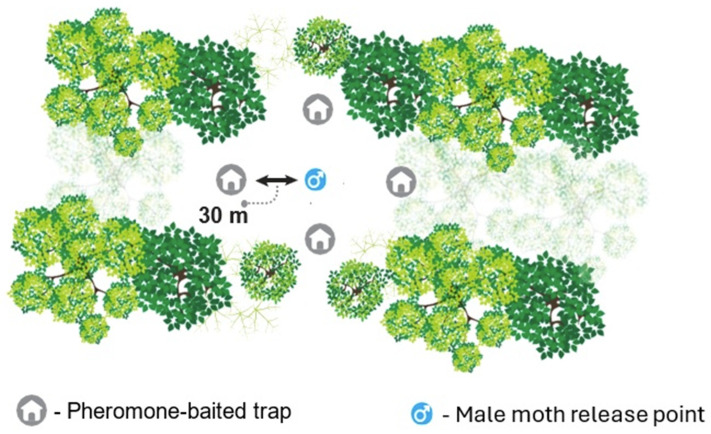
Experimental plot design established to determine the efficacy of mating disruption treatments (figure courtesy of Michael Stamper, Virginia Tech).

The experimental plots were monitored from week 2 to week 10 after the treatment application (6 June–4 August 2023); week 1 was skipped due to logistic constraints. We assessed the efficacy of the applied pheromone treatments according to our standard protocol for the formulation efficacy test using laboratory-reared adult males [25,26]. In each plot, we established a male moth release point surrounded by four USDA milk carton traps suspended approximately 1.5 m above ground within closed-canopy mixed hardwood stands (Figure 4). Each trap was baited with a twine dispenser containing 500 µg of (+)-disparlure, spongy moth sex pheromone (Scentry Biologicals, Inc., Billings, MT, USA), and contained a DDVP strip. Lures were not replaced as the longevity of 500 µg (+)-disparlure dispensers exceeds the duration of the monitoring interval [27].

We released an equal number of spongy moth males per release (50–100) 1–3 times per week for a total of eleven releases. Across the full study period, a total of 610 male moths were released per plot, with recapture rates ranging 1–10.67% per trap per release, consistent with previous mark–release–recapture studies of spongy moth [28]. This spongy moth release rate adequately simulates population densities currently controlled by mating disruption treatments [29]. Mating disruption was evaluated by monitoring male moth recaptures in pheromone-baited traps. Only lab-reared males were used for the analysis to ensure equal population densities among experimental plots. Marked spongy moth males were supplied by the USDA APHIS, Pest Survey Detection and Exclusion Laboratory (Buzzards Bay, MA, USA), and reared to adults in the field lab just prior to release. A solvent red 26 dye (Royce International, Paterson, NJ, USA) was added to the larval diet at the rearing facility. The dye is expressed in adults and is used to differentiate between released and feral male moths. Traps were checked and emptied prior to each moth release.

### 2.3. Efficacy of the Mating Disruption Treatment One Year After the Application

In 2024, we monitored the experimental plots treated in 2023 with SPLAT^®^ SM-O at 14.8 g AI/ha and used the same protocol to evaluate the effect of that treatment on spongy moth mating success one year after the application. In the untreated control plots and the plots treated in 2023, we established a male moth release point surrounded by four pheromone-baited traps and released an equal number of males per release (50–100). We made six male moth releases between 14 June and 31 July 2024. Mating success was evaluated by monitoring male moth recaptures in pheromone-baited traps. Only lab-reared males were used for the analysis to ensure equal population densities among experimental plots.

### 2.4. Statistical Analysis

The data from the plot that received a reduced application rate (11.4 g AI/ha) relative to the target rate (14.8 g AI/ha) were analyzed separately and were not pooled with plots treated at the target application rate. We used a linear mixed-effects model (mixed model ANOVA) to compare trap catches between control and treated plots. Treatment and week were treated as fixed effects, plot was included as a random effect to account for repeated measures and variation among plots, and plot-by-treatment interaction used as an error term. Week was included as a fixed effect to account for temporal variation in background pheromone conditions and phenology. Model fitting was performed using restricted maximum likelihood (REML) (JMP Pro 18, 2025, SAS Institute Inc., Cary, NC, USA). Post-hoc comparisons among treatments were conducted using Tukey’s Honestly Significant Difference (HSD) test. Least squares means and standard errors were extracted for pairwise comparisons.

Given the limited number of treated plots, treatment effects are reported primarily using effect size estimates with associated measures of uncertainty (standard errors) to emphasize the magnitude and uncertainty of responses rather than relying exclusively on null-hypothesis significance testing.

To evaluate overall and weekly percent trap catch reductions, we calculated percent reduction relative to the untreated control using raw means for each treatment group, following Abbott’s formula [30]. In the STS Program, treatment is considered effective if it results in ≥90% reduction in trap catch for at least eight weeks post-application [31] to cover the entire period of spongy moth flight (up to six weeks) and to provide a safety margin for uncertainties associated with the logistics of treatment planning and with spongy moth phenology. Therefore, we used the STS ≤ 10% of control trap catch threshold [31] as a descriptive reference; figures display raw means ± SE to facilitate visual comparison with the program-defined efficacy threshold.

## 3. Results

### 3.1. Efficacy of the Mating Disruption Treatment in the Year of Application

**Figure 5:**
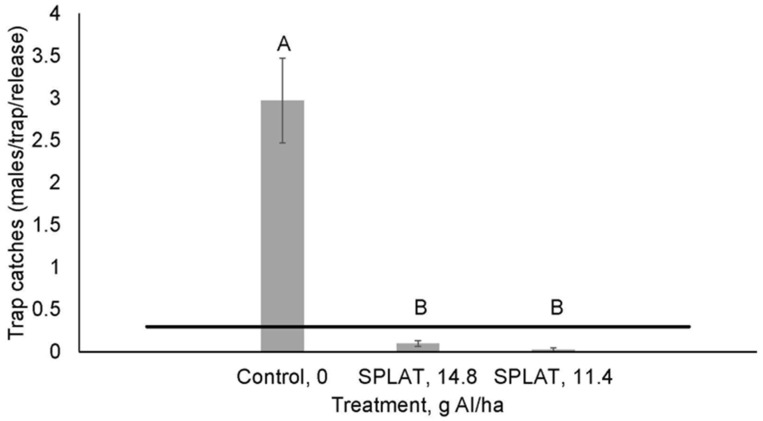
Mean trap catches (±SE) recorded in pheromone-treated and untreated control plots. The black horizontal line represents the 10% mating success threshold used in the STS Program; bars below this line indicate successful treatment. Bars with the same letter are not significantly different (Tukey’s HSD: *p* > 0.05).

Trap catches in the pheromone-treated plots were significantly reduced compared to the untreated control plots (*F*_2,57_ = 599, *p* = 0.003; Figure 5). Across all treated plots, season-long trap catch was reduced by more than 90% relative to the untreated controls. In plots treated with 14.8 g AI/ha (operational dosage), total trap catch was reduced by an average of 97%, ranging from 95% to 99% among plots. Notably, the plot treated with 11.4 g AI/ha (due to a calibration error) also showed a 99% reduction in trap catch.

**Figure 6:**
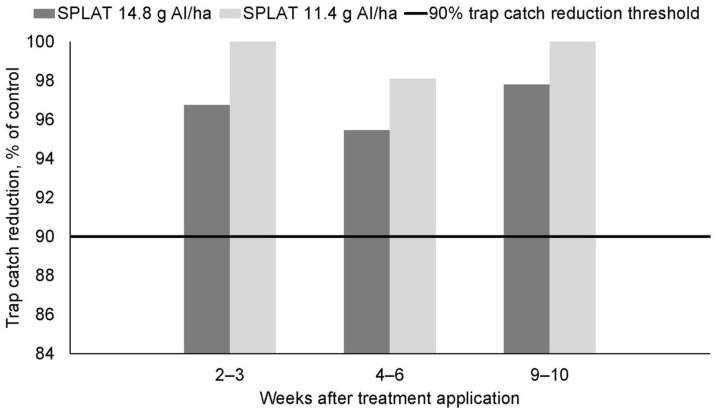
Weekly percent trap catch reduction in plots treated with SPLAT^®^ SM-O at 14.8 g AI/ha (dark gray) and 11.4 g AI/ha (light gray). The black horizontal line represents the 90% trap catch reduction threshold used in the STS Program; bars above this line indicate successful treatment.

Although season-long trap catch was reduced by more than 90%, this metric alone does not indicate whether treatment efficacy was sustained throughout the adult flight period. To evaluate the temporal consistency of treatment efficacy, we analyzed weekly trap catch data. In all treated plots, weekly trap catch was reduced by more than 95% for ten consecutive weeks (Figure 6), confirming that the treatment maintained full efficacy throughout the season.

### 3.2. Efficacy of the Mating Disruption Treatment One Year After the Application

**Figure 7:**
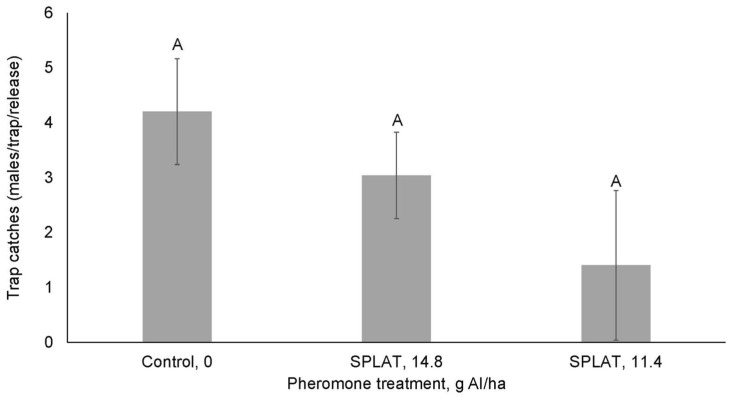
Mean trap catches (±SE) recorded in treated and untreated control plots. Bars sharing the same letter are not significantly different (Tukey’s HSD: *p* > 0.05).

In 2024, one year after the UAV application, season-long trap catches in plots treated with 14.8 g AI/ha and 11.4 g AI/ha were reduced by 28% and 67%, respectively, relative to the untreated control plots. This trap catch reduction one year after the treatment application is comparable to the trap catch reductions observed one year following fixed-wing aerial treatments [32]. Although season-long trap catch reduction varied among the treated plots (2–47% in plots treated with 14.8 g AI/ha dosage and 67% in the plot treated with 11.4 g AI/ha), these differences were not statistically significant (Figure 7).

## 4. Discussion

In this study, we evaluated the in-season efficacy, longevity, and second-year effects of an operational semi-solid, non-Newtonian pheromone formulation applied via unmanned aerial vehicles (UAVs) for mating disruption and interpreted these outcomes in the context of established fixed-wing aerial application methods. The UAV-mounted application system deposits the operational semi-solid pheromone formulation as large, cohesive masses, producing deposition patterns that differ from those generated by conventional fixed-wing applications [33]. These differences have important implications for pheromone release kinetics, persistence, and operational efficacy. Semi-solid pheromone formulations used for mating disruption are designed to release pheromones primarily through diffusion from a stable matrix, resulting in sustained emission over extended periods. Larger deposits have a lower surface-area-to-volume ratio than finely atomized droplets, which is expected to reduce initial burst release and slow pheromone depletion, thereby extending effective field longevity. Previous studies of semi-solid pheromone formulations applied by ground or aerial methods have demonstrated that formulation longevity and efficacy are closely linked to deposit size and structural stability, with larger deposits maintaining disruptive pheromone concentrations for weeks to months [3,33]. The cohesive deposits produced by the UAV-mounted application system in this study are therefore consistent with the intended release characteristics of this formulation class and would not be expected to compromise efficacy.

The sustained ≥95% weekly trap catch reduction observed for ten consecutive weeks in this study supports this interpretation and indicates that UAV-applied deposits, despite differing in physical form from conventional spray applications, provided pheromone release rates sufficient to maintain full seasonal disruption.

From an operational perspective, the ability to place stable, localized deposits may also reduce drift and improve placement precision in forested environments, which is particularly relevant for small or isolated treatment blocks. These characteristics align well with the operational goals of the STS Program, where reliable in-season disruption must be achieved while maintaining compatibility with standard monitoring protocols.

In addition, persistence of pheromone treatments beyond the year of application is an important consideration in STS operations [34]. While residual pheromone may contribute to continued suppression, it can also interfere with trap-based monitoring, as spongy moth pheromone is known to persist in the environment for a year or more. Previous work has shown that droplet size distribution can influence persistence of semi-solid pheromone formulations, with larger droplets releasing pheromones over longer periods. In the present study, UAV application produced fewer, larger cohesive deposits than conventional fixed-wing aerial applications, a pattern that might be expected to enhance second-year effects. However, the modest and variable second-year trap catch reductions observed here indicate that deposit size alone is not a reliable predictor of long-term efficacy. Weathering, canopy interception, application pattern, and environmental conditions may limit persistence even when deposits are large. This finding is consistent with previous studies reporting substantial variability in second-year effects following mating disruption treatments using the same semi-solid pheromone formulation [3,25]. Importantly, the magnitude of second-year reductions observed following UAV application fell within the range reported for conventional fixed-wing aerial treatments [25].

From an STS operational perspective, this similarity is critical. It indicates that UAV-applied pheromone treatment does not fundamentally alter expected second-year outcomes and that existing post-treatment evaluation protocols remain applicable regardless of the application platform. When evaluated using the same trapping protocols, response metrics, and efficacy criteria previously applied to fixed-wing aerial treatments, UAV-applied treatments achieved comparable in-season and second-year performance. Although this study was not designed as a randomized, head-to-head comparison of application platforms, the in-season and second-year efficacy of UAV-applied pheromone treatments were evaluated using the same trapping protocol, response metrics, and efficacy criteria previously used to assess fixed-wing aerial applications of the same semi-solid pheromone formulation [25,35].

In the STS Program, the mean treatment block size for aerially applied mating disruption is large, averaging 2029 ± 113 ha [14]. In contrast, UAVs are typically deployed in much smaller or spatially fragmented blocks, with the capacity to treat approximately 200 ha (500 acres) per day and a minimum operational block size of about 2 ha (5 acres) (Aksata, 2025, pers. Comm.). At current operational rates, treatment costs for UAV applications range from $20 to $60 per 0.4 ha (one acre), depending on site complexity and logistics. Although UAV application costs are comparable to those reported for aerial applications of *Bacillus thuringiensis* var. *kurstaki* (Btk) [14], total treatment costs may be lower for SPLAT^®^-based mating disruption because of lower product costs and the ability to efficiently access treatment areas that would otherwise be difficult or costly to manage using conventional aircraft. Consequently, UAV-based applications may provide an economically viable alternative for small, isolated, or operationally challenging areas where conventional fixed-wing treatments are either impractical or cost-prohibitive.

Accordingly, our findings support the view that UAVs should be integrated as complementary tools within existing forest pest management programs, rather than as replacements for conventional aerial platforms, a conclusion consistent with recent forestry-focused UAV evaluations [11,12]. Integrating UAVs with fixed-wing aircraft in an operational program could maximize budget efficiency and enable treatment of all critical areas, including small, isolated eradication blocks, without leaving untreated gaps or applying excessive coverage. Because UAV deployment is intended for small, isolated, or fragmented blocks rather than for STS-scale aerial treatment blocks, direct per-hectare cost comparisons with fixed-wing operations over large contiguous areas are not informative without a full operational cost model (e.g., ferry time, airspace constraints, and contracting structure).

While a formal cost comparison was beyond the scope of this study, future analyses incorporating explicit operational cost metrics would be valuable for quantifying these trade-offs under different management scenarios.

Many management programs rely on semi-solid, non-Newtonian formulations of semiochemicals, which are currently applied by hand [17,19,20,36], using ground-based equipment [15], or aerially via fixed-wing aircraft [25]. Each of these application methods presents unique operational challenges due to labor, terrain, cost, or logistical constraints. UAV-based delivery could overcome some of these limitations by enabling faster, more uniform, and more precise applications than manual or ground-based methods, and more cost-effective, targeted, and flexible deployment than fixed-wing aerial systems.

By demonstrating that a UAV-applied operational semi-solid spongy moth pheromone formulation meets established performance standards in both the year of treatment and the following season, this study contributes to the growing body of evidence supporting UAVs as practical tools for semiochemical-based pest management and provides a foundation for their integration into operational spongy moth management programs. More broadly, these results suggest that UAV-based delivery systems may have utility in other forest pest management applications, including semiochemical-based approaches targeting pests such as bark beetles, where treatment blocks are small, isolated, spatially fragmented, or otherwise difficult to treat using conventional aircraft.

## 5. Conclusions

This study shows that unmanned aerial vehicle (UAV)-applied mating disruption using an operational semi-solid pheromone formulation achieves in-season and second-year efficacy comparable to established fixed-wing aerial applications when evaluated using standard National Slow the Spread (STS) monitoring protocols. These findings indicate that UAV deployment does not compromise biological performance or post-treatment evaluability and can be integrated into existing STS operational frameworks without requiring modification of assessment criteria.

The principal significance of these results lies in the operational flexibility provided by UAV-based application. UAVs offer a practical means of treating small, isolated, or spatially fragmented forest blocks, including eradication blocks, that are difficult or inefficient to address with fixed-wing aircraft, thereby reducing the likelihood that untreated areas undermine area-wide suppression efforts. UAVs should therefore be viewed as a complementary tool that extends the reach of STS mating disruption rather than as a replacement for conventional aerial treatments.

Although a formal cost comparison was beyond the scope of this study, the comparable performance of UAV-applied and fixed-wing treatments suggests that UAV deployment may be economically viable for targeted applications. Future analyses incorporating explicit operational cost metrics would be valuable for optimizing the integration of UAV-based delivery into spongy moth management programs.

## Data Availability

The raw data supporting the conclusions of this article will be made available by the authors upon request.
